# Micro and Macro-Habitat Associations in Saproxylic Beetles: Implications for Biodiversity Management

**DOI:** 10.1371/journal.pone.0041100

**Published:** 2012-07-25

**Authors:** Joakim Hjältén, Fredrik Stenbacka, Roger B. Pettersson, Heloise Gibb, Therese Johansson, Kjell Danell, John P. Ball, Jacek Hilszczański

**Affiliations:** Department of Wildlife, Fish, and Environmental Studies, Swedish University of Agricultural Sciences, Umeå, Sweden; University Copenhagen, Denmark

## Abstract

Restoration of habitats is critically important in preventing full realization of the extinction debt owed as a result of anthropogenic habitat destruction. Although much emphasis has been placed on macrohabitats, suitable microhabitats are also vital for the survival of most species. The aim of this large-scale field experiment was to evaluate the relative importance of manipulated microhabitats, i.e., dead wood substrates of spruce (snags, and logs that were burned, inoculated with wood fungi or shaded) and macrohabitats, i.e., stand types (clear-cuts, mature managed forests, and forest reserves) for species richness, abundance and assemblage composition of all saproxylic and red-listed saproxylic beetles. Beetles were collected in emergence traps in 30 forest stands in 2001, 2003, 2004 and 2006. More individuals emerged from snags and untreated logs than from burned and shaded logs, but species richness did not differ among substrates. Assemblage composition differed among substrates for both all saproxylics and red-listed saproxylic species, mainly attributed to different assemblage composition on snags. This suggests that the practise of leaving snags for conservation purposes should be complemented with log supplementation. Clear-cuts supported fewer species and different assemblages from mature managed forests and reserves. Neither abundance, nor species richness or assemblage composition differed between reserves and mature managed forests. This suggests that managed stands subjected to selective cutting, not clear-felling, maintain sufficient old growth characteristics and continuity to maintain more or less intact assemblages of saproxylic beetles. Thus, alternative management methods, e.g., continuity forestry should be considered for some of these stands to maintain continuity and conservation values. Furthermore, the significantly higher estimated abundance per ha of red-listed beetles in reserves underlines the importance of reserves for maintaining viable populations of rare red-listed species and as source areas for saproxylic species in boreal forest landscapes.

## Introduction

Human activities have caused substantial fragmentation, loss and degradation of habitats worldwide [1], [2]. In the short-term, many species have survived the initial decrease in habitat availability, but it is likely that an extinction debt is yet to be paid [3], with many species in decline. Without intervention, these species may become extinct. Managed habitats present an opportunity for conservation and production to co-exist through the restoration of microhabitats, which may improve both spatial and temporal connectivity for many species. Conservation of species with specific resource requirements, such as many invertebrates, might be achieved through retaining or restoring microhabitat elements [4], [5], [6]. At a larger scale, managed landscapes present a similar opportunity through the conservation of important macrohabitats, such as old-growth forest. Although many forms of agriculture completely transform landscapes, silvicultural systems often provide the opportunity to retain many elements of the original landscape, so they are more suitable for this blend of conservation and production.

In natural forest ecosystems, structural features such as dead wood are maintained and continuously created through successional processes and natural disturbances, including gap dynamics, storms and forest fires [7]–[9]. This creates a mosaic landscape with a high diversity of both micro and macro-habitats, thus providing suitable living conditions for many different organisms and a continuous input of dead and dying trees. However, modern forestry produces even-aged monocultures with short rotation periods [8], [10]. A consequence is a dramatic decrease in the amount of dead wood in many forest ecosystems, especially large diameter wood and wood in later decay stages [10]–[12]. This has a negative effect on biodiversity because different forms of dead wood are among the most important structural components for maintaining biodiversity in boreal forests [13]–[17]. In addition, many saproxylic (wood living) species are dependent on forest continuity and are therefore negatively affected by clear-felling practices. The number of species directly or indirectly dependent on dead wood is very large, probably between 5000–7000 species in Fennoscandia [17], [18]. In Sweden, approximately 50% or 2131 of all red-listed species are considered forest dependent [19], and ca 60% of these 2131 species are saproxylic.

The importance of stopping and reversing the trend of species loss in forest ecosystems has been widely recognized, and there are general guidelines for biodiversity management of forest areas, commonly based on the need to mimic natural disturbances in forests [20]. In most countries, a combination of increasing the area of protected land, along with conservation measures in the managed landscape, is used to maintain biodiversity. In Sweden, these conservation measures, applied to fulfil legal demands and certification requirements, include: leaving standing and lying dead trees; saving trees along creeks and lakes; creating snags (mainly of Norway spruce, *Picea abies* (L.) Karst.); and burning clear-cut areas [21]. Snags are the substrate most commonly recommended for conservation and restoration for saproxylic species [14], [22], [23] but there are indications that additional substrate types should be provided to maintain biodiversity [5], [24]–[26]. Furthermore, the importance of dead wood retention for maintaining saproxylic biodiversity is poorly understood [27]. Although information on the habitat associations of many species has been published [28], [29], [30], [31], we still lack detailed information for many saproxylic invertebrates, particularly those under threat. Most threatened species occur in low numbers, which makes sampling difficult [32] and targeting of conservation efforts problematic.

The importance of forest reserves for maintaining biodiversity has also been debated. Although old growth reserves are generally regarded as being of high value for conservation, due to high temporal continuity and a high diversity of dead and living trees [16], [17], some studies show that these important features can also be found in managed forests [33]–[35]. It is therefore important to evaluate if there are levels of forestry impact that can be maintained without losses of important structural components and associated species. Thus, it is important to measure not only the importance of forest reserves for threatened saproxylic species, but also the status of these species in managed forests.

We used a large-scale field experiment based on six different types of experimental dead wood substrates, monitored at four occasion over a six year period (2001, 2003, 2004, 2006), to evaluate the micro (substrate) and macro (habitat) associations of saproxylic beetles. The current study is part of a restoration project using a large-scale experimental set up that already has generated several publication (e.g. [12], [34], [35]). However, the results presented here is based on a much larger dataset (all year compared to the single years 2002 or 2003 in the earlier publications) which allows us to address questions not possible in earlier publications, e.g. related to the distribution of red-listed beetles in micro and macro-habitats and the total production of beetles in different macro-habitats. Here, we address the following questions:

1. Are some substrates more important than others for saproxylic species in general and red-listed saproxylic beetles in particular? We ask if snags, the most commonly used substrate for conservation purposes, supports a higher richness and abundance of saproxylic beetles including red-listed beetles but we also evaluate the complementarity of different substrate types.
2. How are saproxylic species including red-listed saproxylic beetles distributed in different types of forest stands? We expect that reserves support a higher species richness and abundance than managed forest, even those only subjected to selective felling.

## Methods

### Study area, experimental design and trapping methods

We conducted our experiment at ten localities in the administrative districts of Västerbotten and Västernorrland within the central-boreal vegetation zone of Sweden [36]. Localities were between the latitudes 63.6208N and 64.2858N and longitudes 16.8898E and 20.1328E, altitude ranged from 100 to 550 m a.s.l. (for further information, see [4]). Each locality consisted of three forest types: an old-growth forest reserve (mean forest age ca 150 yr at the start of the experiment), a mature managed forest (mean age ca 110 yr) and a clear-cut area (Clear-felled in 1999–2000). The age of the mature managed forests is in the upper age range at which forests are clear-felled in northern Sweden, i.e., at age 90–120 according to the Swedish Forest Agency. The total dead wood volumes were ca 11, 15 and 34 m^3^ per ha [37] which is comparable to levels in earlier studies [38]. Distances between our study stands ranged between 5.1 and 101.9 km for clear-cuts, 7.2 and 101.7 km for managed forests, and 7.6 and 103.0 km for old-growth stands. In addition, distances between stands within a locality ranged between 0.2.and 5.6 km with a mean value of 2.2 km. The research areas were Norway spruce-dominated (*Picea abies* Karst.) forests of *Myrtillus*-type understory [39], mainly surrounded by managed forests of a range of age classes. Scots pine (*Pinus sylvestris* L.) was the next-most common tree species, while birch (*Betula* spp.) and aspen (*Populus tremula* L.) also occurred in sparse populations, both in the research areas and surrounding forests.

In each stand, 18 spruce logs and three high stumps were used. The spruce logs all originated from two cuttings of mature forests ca 90–110 years old, one performed early in 2001 (for one locality) and one in autumn 2001 (nine localities). They were placed out at the study stands during early spring 2001 (one locality) and winter 2001–2002, well before the emergence of any saproxylic beetles. The size of the logs was standardized to 4 m in length and 20–25 cm in diameter. We used a randomized block design with three blocks (20–100 m apart) per forest type and locality. Each block (∼15×15 m) consisted of an untreated log (control), a fire-scorched log (scorched *in situ*), an untreated log placed in a naturally shaded place, and two logs inoculated with wood fungi, i.e. *Fomitopsis pinicola* (Swartz ex Fr.) Karst. (Fomitopsidaceae) and *Resinicium bicolor* (Alb. & Schwein) Parmasto (Meruliaceae). We performed the fungi inoculations in situ by drilling three holes with a diameter of 10 mm at four places along the log and filling the holes with mycelium grown on spruce sawdust. Inocula of the fungi were prepared by growing starter cultures on Hagem agar for two weeks and then allowing the fungal strains to ramify through sterilized spruce wood sawdust for at least three weeks. *Fomitopsis pinicola* is a perennial fungus that causes brown rot on both coniferous and deciduous trees. It is one of the most common polypores in the coniferous forest regions of the northern hemisphere [40] and is believed to be important for saproxylic beetles [41]. *Resinicium bicolor* is a white rot and is an early successional species on spruce [42]. The effect of the inoculation was checked one year later by cores taken 10 cm from the place for inoculation on 10 randomly chosen logs for each fungi species. We placed the cores aseptically onto Hagem agar Petri dishes and identified any outgrowing mycelia from hyphal characteristics and back-paired to the original isolates. The inoculations were successful on 54 and 46% of the logs for *F. pinicola* and *R. bicolor*, respectively. This can be regarded as a good result after such a short time period as it usually takes several years for the hyphae to colonise a larger part of a log (J. Stenlid, personal communication). The scorching of the logs was performed with a LPG-burner (liquid petroleum gas) and approximately to the level that commonly seen on burned clear-cuts, i.e. the bark was scorched, but not burned off. The individual logs were randomly assigned to the different treatments in each block. In addition, a spruce snag (3–4 m in height) was created *in-situ* by cutting a living tree in each block in the mature managed forests and reserves. However, in clear-cuts, essentially all trees were harvested and we therefore selected one snag created during the logging operations in each block to correspond to the experimental cut high stumps in the other forest types.

Trunk emergence traps [43]–[46] were used to collect beetles emerging from the experimental dead wood substrates, over four years: June–October 2001 (one locality only); May–September 2003, 2004 and 2006 (all localities). The emergence traps were designed to collect all insects emerging from a 30 cm enclosed section of the logs and snags, by wrapping this section in polypropylene weed barrier cloth, which was kept separated from the log by wire. The traps were sealed with wire at both ends and moved to a new position on the log every trapping year to avoid any influence on the natural colonization or succession by insects. To collect emerging insects, a white 250 ml plastic bottle was attached to the top of the trap and 1/3 filled with 50% ethylene glycol and some detergent to reduce the surface tension.

All collected beetles were identified to species, with the exception of *Acrotrichis* sp. (Fam. Ptiliidae). However, to avoid the influence of “tourists”, i.e. non-saproxylic species, we selected species known to be saproxylic. We defined saproxylic species according to Speight (1989) [47] and also classified beetles according feeding habits using the database for saproxylic beetles [18], to which species confined to the northern part of Sweden were added (Hilszczański, J., Pettersson, R. and Lundberg, S. pers. comm.). The beetles were classified as red-listed based on the Swedish red list [19]. Nomenclature and taxonomy of the beetles follows Lawrence (1995) and Silfverberg (2004) [48], [49].

Dead wood was surveyed in four line transects (100×5 m) for each of the forest types within the ten localities in September 2003 (for details see [37]). Tree species, length/height, and end-diameters within transects were recorded for logs and snags with minimum diameter of 10 cm. The decay class of each dead wood item was also recorded, using a simplified classification system derived from [50] and detailed in [37]. In this study we only used data for dead spruce belonging to decay class DC1 (defined as dead wood with bark intact or starting to loosen, 50% bark remaining, wood hard), as our experimental logs and snags still belonged to this class.

### Statistical analyses

Prior to analyses all data was pooled per stand, for each substrate type individually. ANOVA was used to test for difference in species richness and abundance among spruce substrates, forest types and the interaction between these two factors. This analysis was conducted for all saproxylic species and red-listed saproxylics separately. Tukey tests were used for pair-wise comparison of factors. In addition, when significant interactions occurred, we examined the effect of each factor at each level of the other factor, using simple contrasts which test relationships among cell means [51]. In all of the analyses outlined above, the assumptions of ANOVA were tested with residual plots and in cases of heterogeneous variances the data were log(x+1) transformed prior to analysis.

We calculated the abundance of all saproxylic species and red-listed species per ha as: (*Vnat/Vexp*) * *Aexp,* where *Vnat* = the naturally occurring total volume of spruce dead wood in decomposition class DC1 (standing and laying wood pooled) per ha in each of our experimental stands, *Vexp* = the total volume covered by the emergence traps at each stand (18 traps per stand; each trap covered π*r^2^*h = π*0.125^2^*0.30 = 0.0147 m^3^ resulting in 0.265 m^3^ sampled); *Aexp* = abundance of all saproxylic species or red-listed species in emergence traps in each of our experimental stands. Neither the original data, nor the transformed data fulfilled the requirements of normality and homoscedasticity for parametric tests so we used the non-parametric Quade test [52], with locality as the block factor, for the analyses of dead wood volumes and abundance per ha. All the above analyses were performed on SYSTAT 13 (Systat Software Inc., 2009).

The similarity between beetle communities composition in different substrates and forest types was tested with PERMANOVA (permutational multivariate analysis of variance; [53] using PRIMER (PRIMER-E Ltd., 2007). The assumptions of normality and homoscedasticity are often difficult to fulfil with ecological data and non-parametric methods of analysis based on permutation tests, such as PERMANOVA, are useful alternatives to parametric tests [54], [55]. As for the ANOVA analyses, pooled data per stand were used to examined the effects of substrate and forest type and their interaction on the beetle assemblages. This analysis was again conducted for all saproxylic species and red-listed saproxylics separately. Data were fourth-root transformed in order to reduce weighting of the more abundant species whilst preserving relative abundance information [56]. We used the Bray-Curtis similarity measure, which is not affected by joint absences [57], no standardization, and 4999 permutations of the data. To clarify which species contributed most to the observed differences in beetle assemblages, we used Similarity Percentage Analysis (SIMPER), also on fourth-root transformed data. This is not a test of statistical probabilities *per se*, but a way of conceptualizing what differs between two sets of data: SIMPER calculates the overall percentage contribution that each species makes to the average dissimilarity between two groups and lists the species in decreasing order of their importance in discriminating the two sets of samples [58].

We also produced sample-based rarefaction curves estimates for both total species richness, including species not present in any sample (chao2) and based on all species actually discovered (Sobs) for each forest type to evaluate the completeness of our sampling. PRIMER (PRIMER-E Ltd., 2007) was used to produce the rarefaction curves.

## Results

In total, we collected and identified 78 186 individuals representing 372 saproxylic species, including 212 individuals and 29 species of red-listed saproxylic beetles (0.27% and 7.7% of the total catch, respectively).

### Species richness and abundance

**Figure 1:**
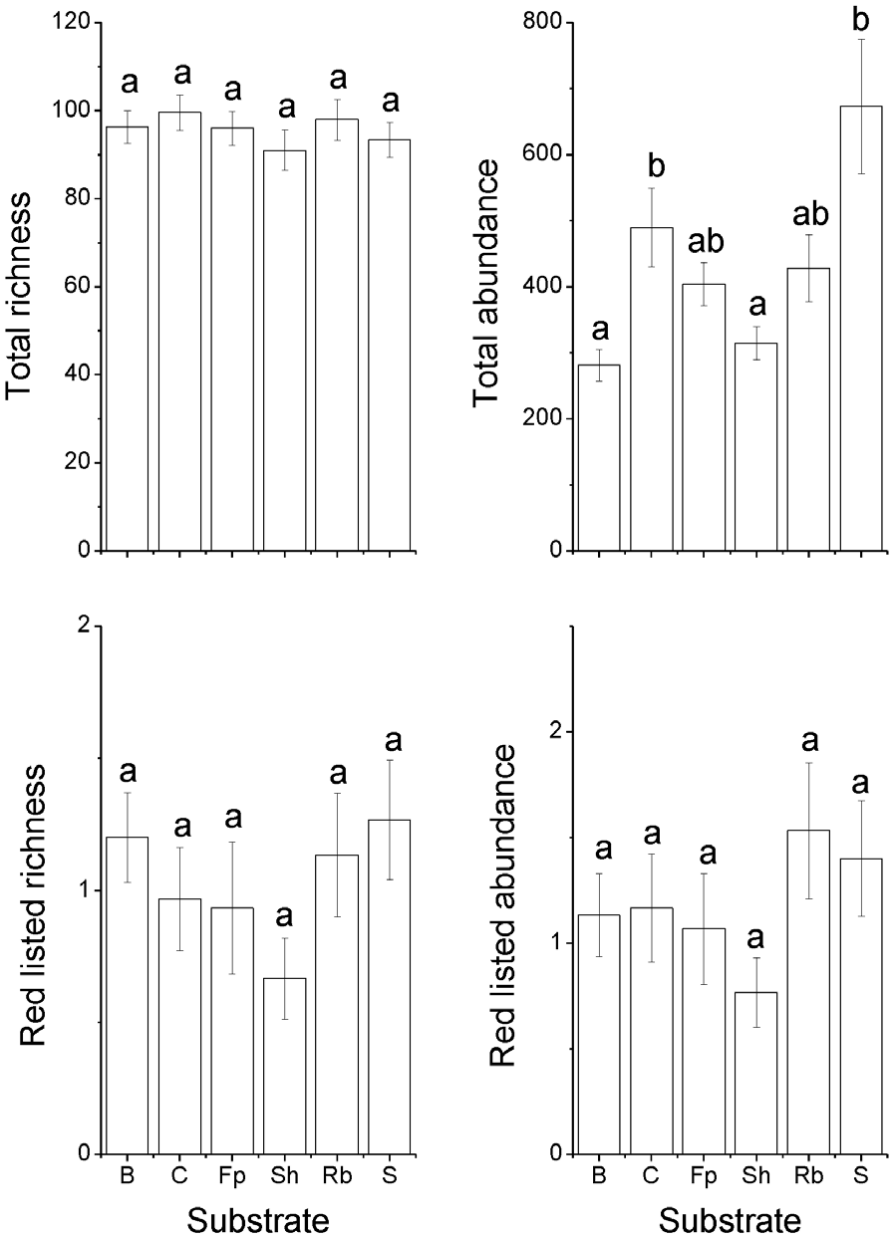
Abundance and species richness in the six different substrate types. Vertical bars shows ±1 SE. Bars with different letters denote statistically significant effects (Tukey test, *p*<0.05). Abbreviations: B = burned, C = control, Fp = *Fomitopsis pinicola*, Rb = *Resinicium bicolor*, Sh = shaded, S = snag. Vertical bars shows ±1 SE.

**Figure 2:**
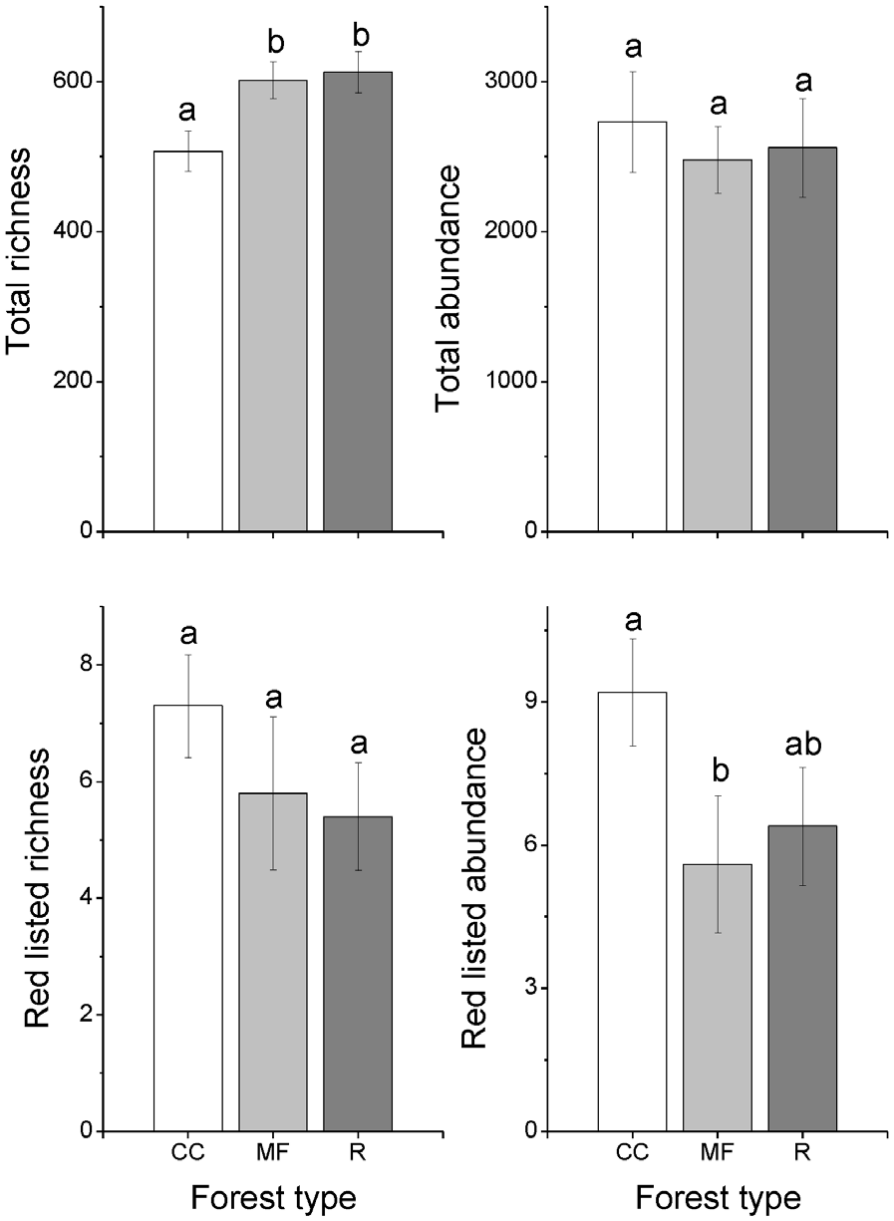
Abundance and species richness in the three different forest types. Abbreviations: CC = Clear-cuts, MF = Mature managed forests, R = Reserves. Bars with different letters denote statistically significant effects (Tukey test, *p*<0.05). Vertical bars show ±1 SE.

**Figure 3:**
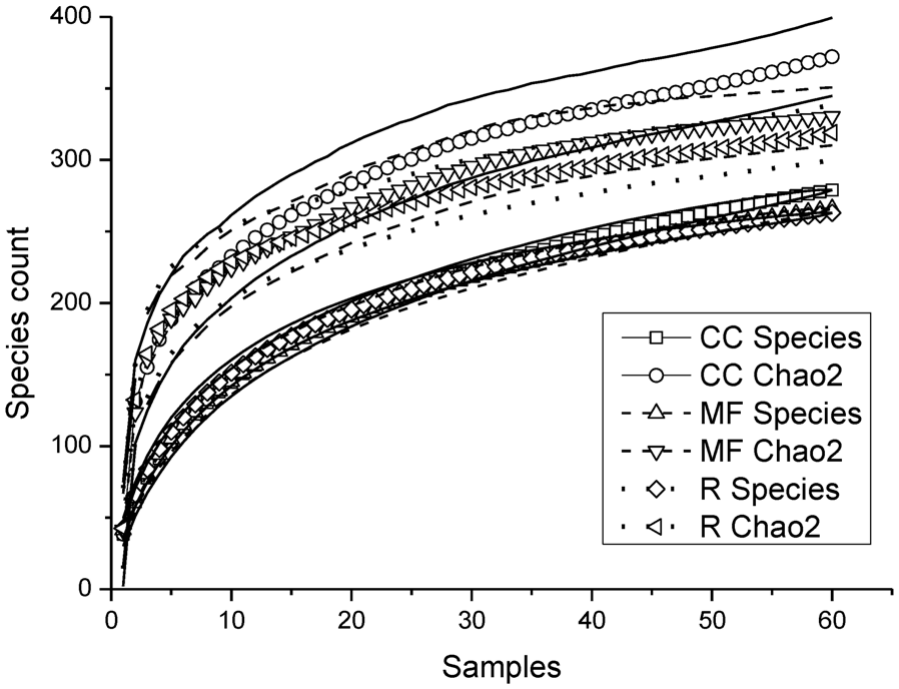
Rarefaction curves. Sample-based rarefaction curves estimates for both total species richness, including species not present in any sample (chao2) and based on all species actually discovered (Sobs) for each forest type. Error curves shows ±1 SD

The total abundance of saproxylic species per stand differed among substrates but not among forest types and there was no significant interaction between these factors (Table 1). Untreated control logs and snags had a significantly higher abundance than burned and shaded logs (Fig. 1). No significant differences were found for the other substrate comparisons, e.g., for fungi inoculated logs. Total species richness differed among forest types but not among substrates. Species richness was lower on clear-cuts than in mature managed forests and reserves (Fig. 2). Neither abundance nor species richness differed among substrates for red-listed species but abundance was higher in clear-cuts than in mature managed forests (Table 1, Fig. 2). The sample based rarefaction curves estimates for both total species richness, including species not present in any sample (chao2) and based on all species actually discovered (Sobs) indicated that species richness in clear-cuts increases more rapidly than in the other substrate types with increasing sample sizes (Fig. 3). This pattern is also indicated by the higher total number of species collected in clear-cuts than in the other forest types (Appendix S2).

**Table 1:** ANOVA and PERMANOVA analyses testing the effect of substrate and forest type on species richness, abundance and assemblage composition of all saproxylic species and red-listed species separately.

Source	df	MS	F	P	Post hoc test
**ANOVA Richness**					
**All saproxylics**					
Substrates	5	0,038	0,650	0,662	
Forest type	2	0,768	13,279	0,000	CC<F, R
Substrate×Forest	10	0,034	0,581	0,827	
Res	162	0,058			
**ANOVAAbundance**					
**All saproxylics**					
Substrates	5	1,567	4,663	0,001	S>B and Sh; C>B
Forest type	2	0,117	0,347	0,707	
Substrate×Forest	10	0,176	0,525	0,871	
Res	162	0,336			
**ANOVA Richness**					
**Red-listed**					
Substrates	5	0,392	1,477	0,200	
Forest type	2	0,459	1,730	0,181	
Substrate×Forest	10	0,039	0,148	0,999	
Res	162	0,265			
**ANOVA Abundance**					
**Red-listed**					
Substrates	5	0,306	0,996	0,422	
Forest type	2	1,236	4,024	0,020	CC>F
Substrate×Forest	10	0,201	0,655	0,765	
Res	162	0,307			

PERMANOVA	df	MS	Pseudo-F	P	Post hoc test
**All saproxylics**					
Substrates	5	6543,4	4,633	0,0002	
Forest type	2	29555	20,926	0,0002	
Substrate×Forest	10	1727,1	1,222	0,0032	CC: Snag # all substrates, B# C and Sh; F and R: Snag #all substrates
Res	162	1412,4			
Total	179				
**Red-listed saproxylics**					
Substrate	5	1480,8	1,508	0,0186	Snags # all other substrates
Forest type	2	5229,5	5,325	0,0002	Clear-cuts # Forest and Reserves
Substrate×Forest	10	871,55	0,887	0,7500	
Res	162	981,96			
Total	179				

When significant interactions occurred, we examined the effect of each factor at each level of the other factor. Tukey tests were used for pair-wise comparisons between substrates (Abbreviations: B = burned, C = control, Fp = *Fomitopsis pinicola*, Rb = *Resinicium bicolor*, Sh = shaded, S = snag) and forest types (CC = clear-cut, F = managed forest, R = reserve)

### Estimated abundance per hectare

**Figure 4:**
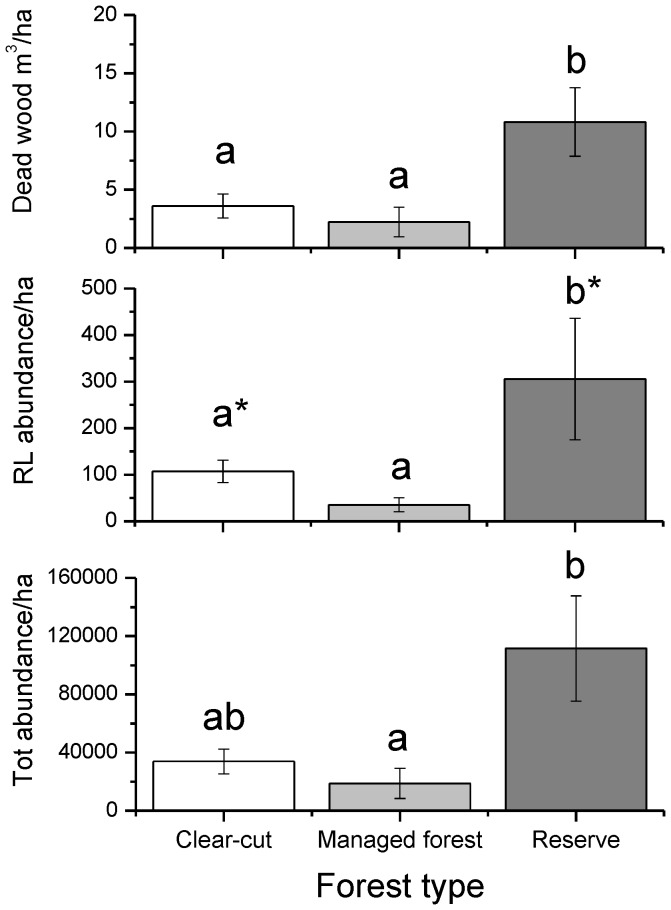
Dead wood volume and abundances/ha. The abundance of dead wood per ha in decomposition class DC1 and the calculated estimated number of individuals produced of red-listed (RL) species and all saproxylic species per ha in the three different forest types. Abbreviations: CC = Clear-cuts, MF = Mature managed forests, R = Reserves. Bars with different letters denote statistically significant effects (Quade multiple comparison test, *p*<0.05, * *p* = 0.098). Vertical bars shows ±1 SE.

The volume of spruce dead wood in decomposition class DC1 (*Vnat*) differed among forest types (Quade Test Statistic: 6.052, p = 0.010) and was significantly higher in reserves than in mature managed forests and clear-cuts (Quade Multiple Comparisons, p = 0.003 and p = 0.048, respectively). The estimated abundance of red-listed beetles in natural dead wood differed among forest types (Quade Test Statistic: 5.639, p = 0.013) and was higher in reserves than in clear-cuts (marginally significant) and mature managed forests (Quade Multiple Comparisons, p = 0.098, p = 0.004, respectively). For all saproxylic beetles, the estimated abundance was higher in reserves than in managed forests (Quade Multiple Comparisons, p = 0. 036) but clear-cuts did not differ significantly from reserves and mature managed forests (Quade Multiple Comparisons, p = 0. 102 and p = 0.590, Fig. 4).

### Assemblages

PERMANOVA revealed that the total assemblage composition differed among forest types and among substrates. In addition, there was a significant interaction between these two factors (Table 1). In clear-cuts, burned substrates supported a different assemblage from shaded logs and controls and snags differed from all other substrates. The species contributing to the differences are listed in Appendix S1. Snag assemblages also differed from all other substrates in both mature managed forest and reserves (Table 1). No other significant differences were found among any of the other substrates (*R. bicolor, F. pinicola*, burned, natural shade and control) in any of the forest types. For red-listed species, patterns were similar, with assemblages differing among both substrates and forest types (Table 1). Snags supported a different assemblage of red-listed species from the other substrate types, among which we found no differences (Table 1). Snags also supported the highest number of unique species of all substrates, ca 13% of the species found on snags was not found on any other substrate. Comparable figures for other substrate ranged between 0 and 6% (Appendix S1).

Clear-cuts had different assemblages from mature managed forests and reserves, regardless of substrate type (Table 1), but mature managed forests and reserves did not differ significantly. The species contributing to the differences are listed in Appendix S2. Furthermore, clear-cuts had a different assemblage of red-listed species from mature managed forests and reserves, whereas assemblages did not differ between mature managed forests and reserves (Table 1). Clear-cuts also supported the highest number of unique species of the forest types, ca 20%, whereas mature managed forests and reserves only had ca 10% unique species each (Appendix S2).

## Discussion

### Micro scale - substrate types

Although, we found that the total abundance of saproxylic beetles was higher on snags than on burned or shaded logs, we found no evidence that snags supported a higher species richness or more red-listed species than the other substrates. Thus, we found no support for the commonly held assumption that spruce snags are a superior substrate for the conservation of saproxylic beetles. Unlike studies using window traps, our results are entirely based on data for saproxylic beetles which actually completed their life cycle in the substrates and were emerging from the wood, and should thus be very reliable. Burned logs produced fewer individuals than control logs and snags, which can be explained by the cambium being partly destroyed by the burning leading to reduced densities of cambium consumers such as dominant bark beetle species (see Appendix S1). This pattern has also been shown in earlier studies [59], [60]. Only one fire specialist (i.e. pyrophilios species sensu [61]) *Stenotrachelus aeneus* was found in our traps and these four individuals were collected on snags. This could also indicate that scorching of individual logs is not sufficient to attract fire specialists. Consistent with this, fire severity has been reported to have a strong influence on the response of pyrophilous beetles [62].

However, abundance and species richness are not the most suitable measures for comparing substrates or habitats, instead guild structure or measurements of species turnover are more efficient and suitable [63]. Snags supported assemblages both of all saproxylics and of red-listed species that were clearly different from the other substrates. This is consistent with earlier studies that reported differences in assemblages of saproxylic insects between standing and lying dead wood [64]–[67].

Interestingly, the difference in assemblages between microhabitats were most pronounced on clear-cuts, where not only snags but also burned and shaded substrates differed from untreated control logs. The most likely explanation is that the effect of shade becomes stronger on the clear-cut because of the high sun exposure compared to mature managed forests and reserves were all substrate types were at least party shaded. As for burned substrates, the species assemblage on clear-cuts is dominated by a small number of highly abundant open habitat substrate specialists. Some of these species are repelled, e.g. *Pityogenes chalcographus* and *Orthotomicus laricis* by burned logs [59]. The lower density of *P. chalcographus* and *O. laricis* on burned logs explained a major part of the difference in assemblages between substrates on the clear-cuts (Appendix S1). It should be noted that the snags on clear-cuts were 1–2 years older (created at clear-felling) than the logs and this could potentially have increased assemblage differences. However, assemblages on snags differed from the other substrates in all forest types suggesting that age difference might have been of minor importance.

Surprisingly, fungal inoculations had no effect on assemblage composition, species richness or abundance of the beetles. Fruiting bodies of *F. pinicola* are known to attract many beetle species [68]. In addition, presence of *F. pinicola* mycelium in dead wood is also strongly correlated with the occurrence of saproxylic beetles [69]. Possible explanations for this lack of inoculation effect are that *F. pinicola* is a common species that colonizes fresh dead wood and it might colonize our substrates regardless if inoculated or not. Inventories of *F. pinicola* fruiting bodies on our experimental substrates revealed slight but non-significant differences between inoculated and control logs, 2.68% and 2.23% had fruiting bodies one year and 12.7% and 10.9% four years after inoculations, respectively (Jörgen Olsson unpublished). This might explain the lack of inoculation effect and suggest that inoculations with this species will not enhance dead wood quality for saproxylic beetles. For *R. bicolor* there are no earlier reports suggesting an association between this species and saproxylic beetles, which also is supported in this study.

The pattern for red-listed species was quite similar to pattern for all saproxylics. Four red-listed species: The fungivorous and detrivorous species *Zilora ferruginea, Ennearthron laricinum, Cis dentatus* and *Bius thoracicus* were consistently more common on snags than the other substrates. In contrast, the predators *Harminius undulatus* and the fungivore *Agathidium nigrinum* were less common on snags (Appendix S1). The clearest pattern was found for *Zilora ferruginea* which is believed to be associated with the wood fungi *Trichaptum abietinum.* Occurrence of *T. abietinum* is a very strong determinant of beetle communities [69]. A previous study suggests that *Z. ferruginea* prefer exposed logs over snags [70], but our data contradicts this, as snags were the preferred substrate for this species.

The clear differences in assemblage composition among dead wood types, particularly with respect to unique species (Appendix S1), highlights the complementarity of substrates. We suggest that in the micro habitat scale, maintenance of a variety of substrate types may be a pre-requisite for maintaining intact assemblages of saproxylic species (see also [26], [65], [71]–[73].In addition to high stumps, it is essential that other dead wood substrates are available to maintain saproxylic species diversity.

### Macro scale - forest types

Forest reserves are considered essential for maintaining biodiversity and function in a managed forest landscape [17], [20], [29], [74]. Stands under low-impact management have been shown to support higher species richness than forests subjected to more intensive forestry [74]–[77] and we therefore expected reserves to support the highest species richness and abundance of saproxylic beetles. However, we only found partial support for this. Although total species richness was higher in reserves and managed forests than in clear-cuts, reserves did not differ from mature managed forests.

The lack of clear distinction between the mature managed forests and reserves could partly be due to the fact that the mature managed stands in this study have never been subjected to modern forestry, only selective felling, because clear-felling was not widely used in Sweden before the 1950s. Thus, a large majority of the mature forests in this area have never been clear-felled, making our forests representative for forest in the area. Earlier studies on other organism groups, e.g., lichens and mosses, as well as saproxylic beetles in general, have also revealed a relatively high species richness and occurrence of many red-listed species in mature managed forests [4], [33], [78]. Thus, managed stands that have historically been subjected only to selective felling have seemingly maintained sufficient levels of important structural components, i.e., dead wood diversity and density (see [37]), as well as forest continuity to maintain more or less intact assemblages of saproxylic beetles. These types of stands constitute 20–25% of productive forest area in the counties included in this study and therefore have the potential to play a very important role for biodiversity in this managed landscape. These forests represent an alternative management strategy that seems to favour diversity. This clearly suggests that continuous cover forestry should be considered as an alternative to clear-felling for future management of these stands.

It should be noted that our study deals with early stages of wood decay and dead wood of modest diameter. For saproxylic beetles associated with later stages of wood decay the distribution pattern in different forest types might be different. However, Stenbacka et al. [12] sampled the same stands with window traps, which should collect saproxylics from all decay stages, but detected no difference in assemblages between mature managed forests and reserves. Although our mature managed forest stands appear old, rotation periods in the study area range from ca 90–120 years. In the counties of Västernorrland and Västerbotten, 25 and 20%, respectively of the productive non-reserve forests are more than 101 years old (www.skogsstyrelsen.se/statistics, table 3.4), making our forest quite representative for the forest in this area.

One important question for our study is the relative importance of local stand quality versus local source community structure, i.e., if the different stands were so close that the local beetle community had a stronger influence on the assemblages colonizing our substrates than the stand conditions. The average distance between different forest stands within a locality was 2.2 km, a distance within the dispersal range of some but far from all saproxylic beetles [79], [80]. It is therefore difficult to answer this question but Stenbacka et al. [37], using window traps, found significant differences in beetles assemblages between younger stands and the mature managed stands in the localities used in this study. This suggests that stand quality is important for our observed patterns.

Despite the similarities in species richness and abundance of saproxylic beetles in reserves and mature managed forests, there are still several strong arguments for the importance of reserves in the managed landscape. Our calculations of abundances per ha suggest that reserves most likely are important as source populations of threatened species at a landscape scale. Our calculations are based on dead wood in the first decay stage but the difference in dead wood volumes and volumes of large diameter deadwood between reserves and other forest types in our study areas are much more pronounced for later decay stages [37]. This means that the difference in abundance between reserves and the other forest types should be expected to be even more pronounced in later decay stages. Penttilä et al. [74] found that very high levels of dead wood, more than 100 m^3^/ha, are needed to maintain intact assemblages of red-listed polypores. Such levels are usually only reached in protected old-growth forests. In agreement with this, the species richness and abundance of red-listed saproxylic wood fungi is higher in the reserves than in the mature managed forests used in our study [81].

However, it should be stressed that our calculation of abundance per ha in different forest types is an approximation. Significant error may result from factors we have not accounted for, e.g., variation in abundances of different species in different substrate types. However, our calculation provides a valuable approximation showing general patterns in the production of saproxylic beetles in different forest types.

The importance of reserves suggested by productivity estimates is also supported by the results from window traps sampling of the same research areas as in this study [37]. More individuals and species, including red-listed species, were collected in reserves than in stands regeneration after clear-felling, regardless of their age [37].Thus, although this study revealed no differences between forest types in the individuals produced on a per volume substrate basis, the much higher volume and diversity of dead wood substrates most likely produces more species and individuals per ha in the reserves as our calculations also imply. These results suggest that, based on the simple measures of abundance and species richness, old growth reserves are important for saproxylic biodiversity (see also e.g. [17], [20], [74], [77]).

The clear differences in assemblages between clear-cuts and the other forest types in assemblage composition were expected (also supported by the high occurrence of unique species in clear-cuts; Appendix S2), as clear-cuts should attract more species associated with early successional forests (e.g. [72], as well as fire [81]; Appendix S2). Our results confirm earlier results that several species, e.g. the cambium consumers *Pityogenes chalcographus, Orthotomicus laricis* and *Crypturgus subcribrosus* and the predator *Ampedus tristis* clearly prefer clear-cuts whereas others, such as *Hylurgops glabratus* and *Trypodendron lineatum* mainly dwell in closed forests [82] (Appendix S2).

The predatory species *Platysoma minus* and *Lacon fasciatus* were only found on clear-cuts and contributed substantially to the assemblage differences for red-listed species (Appendix S2). In contrast, the fungivores *Agathidium mandibulare, Atomaria alpina* and *Olisthaerus substriatus* only occurred in closed forest habitats and they also contributed significantly to the assemblage differences among forest types (Appendix S2). *A. alpina* has also shown a significant and positive association to the abundance of fruiting bodies of the wood fungi *Fomitopsis pinicola,* probably because it eats *F. pinicola* spores [83].

In conclusion, both micro- and macro-habitat can be managed to enhance saproxylic beetle diversity. We found no support for snags being a superior substrate for management of saproxylic biodiversity. Instead, our results suggest that snags should be complemented with different log substrates to provide substrate for the wide diversity of saproxylic beetles. Our results on macrohabitat level suggest that all forest types are important for maintaining biodiversity in a managed forest landscape. As large scale disturbances such as fire are becoming increasingly rare, it is important to provide a variety of microhabitats for saproxylics in clear-cuts as this habitat attracts species normally associated with fire disturbance. However, as old growth forests are rare in the forest landscape (forests older than 141 years constitute only 6.7% whereas forest 0–30 years constitutes 33% of Swedish forest landscape), reserves are most likely to play an important role both as lifeboats for maintaining population of rare old-growth associated species and as source populations for colonization of restored forest habitats at a landscape scale. Although mature managed forest produced less saproxylic beetles per ha than reserves, they were equally species rich and supported similar assemblages to reserves. This indicates that they still maintain sufficient old growth characteristics and to contribute to landscape conservation value. Alternative management methods should be considered for these stands to maintain forest continuity and restore microhabitats for saproxylic species.

## Supporting Information

Appendix S1 **Saproxylic beetles caught in the eclector trap in different substrate types, where B = burned log, L = untreated log, Fp =** ***Fomitopsis pinicola*** **inoculated log, Rb =** ***Resinicium bicolour*** **inoculated log, Sh = shaded log, and Sn = snag.** The results from the Simper analyses (Simp) are presented both for all species, separated by forest types (CC = Clear-cut, F = forest, R = reserve) due to a significant interaction term in Table 1, and red-listed species, not separated by forest type due to lack of an significant interaction term in Table 1. The 10 species that contributed most to the differences (calculated as the mean contribution for all significant comparisons in the Simper analysis) in assemblages between substrates are ranked in descending order (e.g. 1 explained most of the variation in assemblages). Rank for all species are followed by forest type (e.g. 1CC) and rank for red-listed species are followed by RL (e.g. 1RL). The wood association status (Stat) (SO = obligate saproxylics and SF = facultative saproxylics) is according to the saproxylic data base (www.saproxylic.org) The red-list categories (RL) are according to Gärdenfors (2010), EN = endangered, VU = vulnerable, NT = near threatened, DD = data deficient.
(DOC)

Appendix S2 **Saproxylic beetles caught in emergence traps in the different forest types, where CC = clear-cut, For = mature forest, and Res = reserve.** The results from the Simper analyses (Simp) presented both for all species and red-listed (RL) separately. The 10 species that contributed most to the differences (calculated as the mean contribution for all significant comparisons in the Simper analysis) in assemblages between forest types (see Table 1) are ranked in descending order (e.g. 1 explained most of the variation in assemblages). Rank for red-listed species are followed by RL (e.g. 1RL). The red-list categories are according to Gärdenfors (2010), EN = endangered, VU = vulnerable, NT = near threatened, DD = data deficient. The wood association status (SO = obligate saproxylics and SF = facultative saproxylics) is according to the saproxylic data base (www.saproxylic.org).
(DOC)
