# Supplementary material for: Micro and Macro-Habitat Associations in Saproxylic Beetles: Implications for Biodiversity Management
Source: PLoS One. 2012 Jul 25;7(7):e41100. doi: 10.1371/journal.pone.0041100 (PMC3405078; doi:10.1371/journal.pone.0041100)
Supplement: Appendix S2 — Saproxylic beetles caught in emergence traps in the different forest types, where CC = clear-cut, For = mature forest, and Res = reserve. The results from the Simper analyses (Simp) presented both for all species and red-listed (RL) separately. The 10 species that contributed most to the differences (calculated as the mean contribution for all significant comparisons in the Simper analysis) in assemblages between forest types (see Table 1) are ranked in descending order (e.g. 1 explained most of the variation in assemblages). Rank for red-listed species are followed by RL (e.g. 1RL). The red-list categories are according to Gärdenfors (2010), EN = endangered, VU = vulnerable, NT = near threatened, DD = data deficient. The wood association status (SO = obligate saproxylics and SF = facultative saproxylics) is according to the saproxylic data base (www.saproxylic.org). (DOC) [file pone.0041100.s002.doc]

**Appendix S2.** Saproxylic beetles caught in emergence traps in the different forest types, where CC = clear-cut, For = mature forest, and Res = reserve. The results from the Simper analyses (Simp) presented both for all species and red-listed (RL) separately. The 10 species that contributed most to the differences (calculated as the mean contribution for all significant comparisons in the Simper analysis) in assemblages between forest types (see Table 1) are ranked in descending order (e.g. 1 explained most of the variation in assemblages). Rank for red-listed species are followed by RL (e.g. 1RL). The red-list categories are according to Gärdenfors (2010), EN = endangered, VU = vulnerable, NT = near threatened, DD = data deficient. The wood association status (SO = obligate saproxylics and SF = facultative saproxylics) is according to the saproxylic data base (www.saproxylic.org).

| **Species name** | **Simp** | **Stat** | **RL** | **CC** | **For** | **Res** | **Total** |
| --- | --- | --- | --- | --- | --- | --- | --- |
| *Abdera flexuosa* |  | SO |  | 1 |  | 1 | 2 |
| *Abdera triguttata* |  | SO |  | 20 | 7 | 4 | 31 |
| *Absidia rufotestacea* |  | SO |  | 3 |  |  | 3 |
| *Absidia schoenherri* |  | SO |  | 22 | 58 | 28 | 108 |
| *Acidota crenata* |  | SF |  | 11 | 34 | 45 | 90 |
| *Acrulia inflata* |  | SF |  | 6 | 29 | 30 | 65 |
| *Agathidium arcticum* |  | SF |  |  | 3 | 1 | 4 |
| *Agathidium laevigatum* |  | SF |  |  | 1 |  | 1 |
| *Agathidium mandibulare* |  | SF | NT |  | 2 | 2 | 4 |
| *Agathidium nigrinum* | 5RL | SF | NT | 2 | 6 | 2 | 10 |
| *Agathidium nigripenne* |  | SF |  |  | 7 | 2 | 9 |
| *Agathidium pallidum* |  | SF | NT | 1 | 1 |  | 2 |
| *Agathidium pisanum* |  | SO |  | 2 | 34 | 32 | 68 |
| *Agathidium rotundatum* |  | SF |  | 10 | 6 | 15 | 31 |
| *Agathidium seminulum* |  | SF |  | 5 | 22 | 22 | 49 |
| *Ampedus balteatus* |  | SO |  | 14 |  | 1 | 15 |
| *Ampedus nigrinus* |  | SO |  | 100 | 4 | 2 | 106 |
| *Ampedus tristis* | 3 | SO |  | 545 | 1 | 16 | 562 |
| *Anaspis arctica* |  | SO |  | 1 |  |  | 1 |
| *Anaspis bohemica* |  | SO |  | 31 |  |  | 31 |
| *Anaspis marginicollis* |  | SO |  | 124 | 52 | 44 | 220 |
| *Anaspis rufilabris* |  | SO |  | 46 | 15 | 49 | 110 |
| *Anisotoma aillaris* |  | SO |  | 19 | 1 |  | 20 |
| *Anisotoma castanea* |  | SO |  | 10 | 6 | 7 | 23 |
| *Anisotoma glabra* |  | SO |  | 10 | 4 | 1 | 15 |
| *Anisotoma humeralis* |  | SO |  | 4 | 12 | 6 | 22 |
| *Anobium rufipes* |  | SO |  | 1 |  |  | 1 |
| *Anobium thomsoni* |  | SO |  | 3 |  |  | 3 |
| *Anomognathus cuspidatus* |  | SO |  | 1 |  |  | 1 |
| *Anopleta corvina* |  | SF |  |  |  | 2 | 2 |
| *Anthaia quadripunctata* |  | SO |  | 3 |  |  | 3 |
| *Anthophagus caraboides* |  | SF |  | 2 | 1 | 1 | 4 |
| *Arpedium quadrum* |  | SF |  | 3 |  |  | 3 |
| *Arpidiphorus orbiculatus* |  | SF |  |  | 16 | 10 | 26 |
| *Asemum striatum* |  | SO |  | 1 |  |  | 1 |
| *Atheta sg Alaobia sodalis* |  | SF |  | 21 | 11 | 10 | 42 |
| *Atheta sg Atheta acutangula* |  | SF |  | 1 | 28 | 21 | 50 |
| *Atheta sg Atheta allocera* |  | SF |  |  |  | 1 | 1 |
| *Atheta sg Atheta aquatica* |  | SF |  | 1 |  |  | 1 |
| *Atheta sg Atheta atramentaria* |  | SF |  | 1 |  |  | 1 |
| *Atheta sg Atheta brunneipennis* |  | SF |  | 1 |  | 1 | 2 |
| *Atheta sg Atheta crassicornis* |  | SF |  | 1 | 3 | 4 | 8 |
| *Atheta sg Atheta euryptera* |  | SF |  | 1 | 2 | 9 | 12 |
| *Atheta sg Atheta harwoodi* |  | SF |  |  | 1 | 2 | 3 |
| *Atheta sg Atheta hypnorum* |  | SF |  | 1 | 3 | 2 | 6 |
| *Atheta sg Atheta incognita* |  | SF |  | 1 | 9 |  | 10 |
| *Atheta sg Atheta nigricornis* |  | SF |  | 4 | 7 | 9 | 20 |
| *Atheta sg Atheta paracrassicornis* |  | SF |  | 1 | 4 | 3 | 8 |
| *Atheta sg Atheta pilicornis* |  | SF |  | 81 | 337 | 305 | 723 |
| *Atheta sg Atheta procera* |  | SF |  | 1 |  |  | 1 |
| *Atheta sg Dimetrota intermedia* |  | SF |  |  | 1 |  | 1 |
| *Atheta sg Microdota subtilis* |  | SF |  | 4 | 16 | 10 | 30 |
| *Atheta sg Traumoecia picipes* |  | SF |  | 7 | 9 | 11 | 27 |
| *Atheta sg Xenota myrmecobia* |  | SF |  | 3 | 7 | 5 | 15 |
| *Athous subfuscus* |  | SF |  | 20 | 10 | 14 | 44 |
| *Atomaria alpina* |  | SO | NT |  | 5 | 2 | 7 |
| *Atomaria atrata* |  | SF |  | 7 | 8 | 4 | 19 |
| *Atomaria badia* | 7RL | SO | NT | 2 | 1 | 4 | 7 |
| *Atomaria bella* |  | SO |  | 202 | 141 | 143 | 486 |
| *Atomaria bescidica* |  | SF |  |  | 1 | 3 | 4 |
| *Atomaria fuscata* |  | SF |  |  |  | 1 | 1 |
| *Atomaria lapponica* |  | SO | NT | 1 | 2 | 8 | 11 |
| *Atomaria ornata* |  | SF |  | 19 | 7 | 12 | 38 |
| *Atomaria peltata* |  | SF |  | 17 | 9 | 8 | 34 |
| *Atomaria procerula* |  | SF |  | 4 | 4 |  | 8 |
| *Atomaria pulchra* |  | SF |  | 33 | 121 | 98 | 252 |
| *Atomaria pusilla* |  | SF |  | 2 | 1 |  | 3 |
| *Atomaria subangulata* |  | SO |  | 137 | 51 | 84 | 272 |
| *Atomaria testacea* |  | SF |  | 1 |  |  | 1 |
| *Atomaria turgida* |  | SF |  | 2 |  |  | 2 |
| *Atomaria umbrina* |  | SO |  | 1 | 10 | 4 | 15 |
| *Atomaria zetterstedti* |  | SF |  |  |  | 1 | 1 |
| *Atrecus affinis* |  | SO |  | 3 | 18 | 12 | 33 |
| *Atrecus longiceps* |  | SO |  | 1 | 15 | 9 | 25 |
| *Atrecus pilicornis* |  | SO |  | 3 | 93 | 65 | 161 |
| *Autalia impressa* |  | SF |  |  | 2 |  | 2 |
| *Bibloporus bicolor* |  | SO |  | 14 | 23 | 28 | 65 |
| *Bibloporus minutus* |  | SO |  | 2 | 3 |  | 5 |
| *Bius thoracicus* |  | SO | VU | 1 |  | 1 | 2 |
| *Bolitochara mulsanti* |  | SO |  |  | 5 | 6 | 11 |
| *Bolitochara pulchra* |  | SF |  | 19 | 7 | 16 | 42 |
| *Bryoporus cernuus* |  | SF |  | 1 |  |  | 1 |
| *Bryoporus crassicornis* |  | SF |  | 1 |  |  | 1 |
| *Bryoporus punctipennis* |  | SF |  |  | 9 | 6 | 15 |
| *Buprestis rustica* |  | SO |  | 2 |  |  | 2 |
| *Caenoscelis ferruginea* |  | SF |  |  |  | 2 | 2 |
| *Caenoscelis sibirica* |  | SF |  |  |  | 3 | 3 |
| *Callidium coriaceum* |  | SO |  | 7 |  |  | 7 |
| *Cardiophorus ruficollis* |  | SO |  | 1 |  |  | 1 |
| *Cerylon ferrugineum* |  | SO |  | 8 | 17 | 11 | 36 |
| *Cerylon histeroides* |  | SO |  | 56 | 41 | 56 | 153 |
| *Cerylon sp.* |  | SO |  |  | 4 |  | 4 |
| *Choleva fagniezi* |  | SF |  | 1 |  |  | 1 |
| *Chrysobothris chrysostigma* |  | SO |  | 5 |  |  | 5 |
| *Cis alter* |  | SO |  |  | 1 |  | 1 |
| *Cis bidentatus* |  | SO |  |  | 4 | 1 | 5 |
| *Cis boleti* |  | SO |  | 3 | 2 | 2 | 7 |
| *Cis comptus* |  | SO |  | 3 | 2 | 1 | 6 |
| *Cis dentatus* |  | SO | NT | 1 | 2 |  | 3 |
| *Cis glabratus* |  | SO |  |  | 2 | 5 | 7 |
| *Cis hispidus* |  | SO |  | 2 | 3 | 3 | 8 |
| *Cis lineatocribratus* |  | SO |  | 3 | 4 | 14 | 21 |
| *Cis punctulatus* |  | SO |  | 14 | 11 | 26 | 51 |
| *Corticaria abietorum* |  | SF |  | 3 | 9 | 4 | 16 |
| *Corticaria fennica* |  | SO | VU |  |  | 1 | 1 |
| *Corticaria foveola* |  | SF |  |  | 4 | 1 | 5 |
| *Corticaria interstitialis* | 1RL | SF | NT | 16 | 5 | 3 | 24 |
| *Corticaria lateritia* |  | SO |  | 3 | 2 | 7 | 12 |
| *Corticaria longicollis* |  | SF |  | 11 | 5 | 5 | 21 |
| *Corticaria orbicollis* |  | SO |  | 9 | 7 | 6 | 22 |
| *Corticaria polypori* | 7RL | SO | NT | 4 | 1 | 6 | 11 |
| *Corticaria rubripes* |  | SF |  | 74 | 16 | 13 | 103 |
| *Corticaria serrata* |  | SF |  | 1 |  |  | 1 |
| *Corticarina fuscula* |  | SF |  | 11 | 1 | 2 | 14 |
| *Corticarina latipennis* |  | SF |  |  | 2 |  | 2 |
| *Corticarina obfuscata* |  | SF |  | 3 | 7 | 2 | 12 |
| *Corticarina similata* |  | SF |  |  | 3 |  | 3 |
| *Corticeus linearis* |  | SO |  | 31 |  |  | 31 |
| *Corticeus suturalis* |  | SO | NT |  |  | 1 | 1 |
| *Cortinicara gibbosa* |  | SF |  | 4 |  |  | 4 |
| *Coryphium angusticolle* |  | SF |  | 4 |  | 8 | 12 |
| *Cryphalus saltuarius* |  | SO |  |  | 1 | 3 | 4 |
| *Cryptolestes abietis* |  | SO |  |  | 1 | 2 | 3 |
| *Cryptophagus abietis* |  | SF |  | 3 | 21 | 8 | 32 |
| *Cryptophagus badius* |  | SF |  | 1 |  |  | 1 |
| *Cryptophagus dentatus* |  | SF |  |  | 2 | 6 | 8 |
| *Cryptophagus lapponicus* |  | SF |  | 2 | 6 | 4 | 12 |
| *Cryptophagus longitarsis* |  | SF |  |  | 10 | 6 | 16 |
| *Cryptophagus pilosus* |  | SF |  | 1 |  |  | 1 |
| *Cryptophagus scanicus* |  | SF |  |  | 4 | 2 | 6 |
| *Cryptophagus setulosus* |  | SF |  | 3 | 3 | 3 | 9 |
| *Crypturgus cinereus* |  | SO |  | 23 | 25 | 30 | 78 |
| *Crypturgus hispidulus* |  | SO |  | 13 | 17 | 45 | 75 |
| *Crypturgus pusillus* |  | SO |  | 2282 | 3907 | 7814 | 14003 |
| *Crypturgus subcribrosus* | 4 | SO |  | 4746 | 3749 | 1600 | 10095 |
| *Curtimorda maculosa* | 9 | SO |  | 487 | 4 | 5 | 496 |
| *Dacne bipustulata* |  | SO |  | 19 | 2 | 1 | 22 |
| *Dadobia immersa* |  | SO |  | 41 | 23 | 29 | 93 |
| *Dalotia coriaria* |  | SF |  |  |  | 1 | 1 |
| *Dasytes niger* |  | SO |  | 60 |  |  | 60 |
| *Deliphrum tectum* |  | SF |  | 4 | 1 |  | 5 |
| *Dendroctonus micans* |  | SO |  | 2 | 1 |  | 3 |
| *Dendrophagus crenatus* |  | SO |  | 2 | 7 | 4 | 13 |
| *Dendrophilus pygmaeus* |  | SF |  | 1 | 1 |  | 2 |
| *Denticollis linearis* |  | SO |  | 3 | 2 | 4 | 9 |
| *Dermestes lardarius* |  | SF |  | 1 |  |  | 1 |
| *Dictyoptera aurora* |  | SO |  | 1 | 3 | 6 | 10 |
| *Dienerella elongata* |  | SF |  |  | 1 |  | 1 |
| *Dienerella filum* |  | SF |  | 10 | 9 | 18 | 37 |
| *Dinaraea aequata* |  | SO |  | 5 | 2 | 6 | 13 |
| *Dinaraea angustula* |  | SF |  |  |  | 1 | 1 |
| *Dinaraea arcana* |  | SO |  | 5 | 11 | 9 | 25 |
| *Dinaraea linearis* |  | SO |  | 1 | 13 | 16 | 30 |
| *Dorcatoma dresdensis* |  | SO |  | 1 |  |  | 1 |
| *Dryocoetes alni* |  | SO |  |  | 1 |  | 1 |
| *Dryocoetes autographus* | 10 | SO |  | 1593 | 5931 | 5377 | 12901 |
| *Dryocoetes hectographus* |  | SO |  | 3 | 38 | 18 | 59 |
| *Endomychus coccineus* |  | SO |  | 3 |  |  | 3 |
| *Enicmus apicalis* |  | SO | NT |  | 2 |  | 2 |
| *Enicmus fungicola* |  | SO |  | 7 | 1 | 1 | 9 |
| *Enicmus planipennis* |  | SO | NT |  |  | 2 | 2 |
| *Enicmus rugosus* |  | SO |  | 89 | 11 | 16 | 116 |
| *Ennearthron cornutum* |  | SO |  |  |  | 1 | 1 |
| *Ennearthron laricinum* | 8RL | SO | NT | 1 | 5 | 3 | 9 |
| *Episernus angulicollis* |  | SO |  |  | 1 |  | 1 |
| *Epuraea aestiva* |  | SF |  |  |  | 1 | 1 |
| *Epuraea angustula* |  | SO |  | 64 | 96 | 103 | 263 |
| *Epuraea biguttata* |  | SO |  | 2 | 1 | 9 | 12 |
| *Epuraea boreella* |  | SO |  | 61 | 143 | 195 | 399 |
| *Epuraea contractula* |  | SO |  |  | 3 | 2 | 5 |
| *Epuraea laeviuscula* |  | SO |  | 7 | 39 | 74 | 120 |
| *Epuraea longipennis* |  | SO |  | 16 | 72 | 83 | 171 |
| *Epuraea longula* |  | SO |  |  |  | 1 | 1 |
| *Epuraea marseuli* |  | SO |  | 5 | 7 | 16 | 28 |
| *Epuraea oblonga* |  | SO | NT |  | 1 |  | 1 |
| *Epuraea opalizans* |  | SO |  |  | 4 | 2 | 6 |
| *Epuraea pallescens* |  | SO |  | 1 | 3 | 5 | 9 |
| *Epuraea pygmaea* |  | SO |  | 24 | 151 | 163 | 338 |
| *Epuraea rufomarginata* |  | SF |  | 3 | 1 | 3 | 7 |
| *Epuraea thoracica* |  | SO |  |  |  | 2 | 2 |
| *Epuraea unicolor* |  | SF |  |  | 2 | 1 | 3 |
| *Epuraea variegata* |  | SO |  | 20 | 5 | 13 | 38 |
| *Ernobius explanatus* |  | SO |  | 1 |  | 7 | 8 |
| *Euconnus claviger* |  | SF |  |  | 1 |  | 1 |
| *Euglenes pygmaeus* |  | SO |  | 29 | 2 |  | 31 |
| *Euplectus decipiens* |  | SO |  |  | 1 | 3 | 4 |
| *Euplectus karsteni* |  | SF |  | 5 |  | 4 | 9 |
| *Euplectus nanus* |  | SF |  | 2 |  | 1 | 3 |
| *Euplectus piceus* |  | SF |  | 1 | 1 |  | 2 |
| *Euplectus punctatus* |  | SO |  | 27 | 38 | 37 | 102 |
| *Euryusa castanoptera* |  | SO | NT |  |  | 1 | 1 |
| *Eutheia linearis* |  | SF |  | 9 |  |  | 9 |
| *Gabrius expectatus* |  | SF |  | 31 | 31 | 48 | 110 |
| *Gabrius splendidulus* |  | SF |  | 1 | 2 |  | 3 |
| *Glischrochilus hortensis* |  | SF |  | 3 | 1 | 2 | 6 |
| *Glischrochilus quadripunctatus* |  | SO |  | 22 | 6 | 3 | 31 |
| *Globicornis emarginata* |  | SO |  | 8 |  |  | 8 |
| *Gyrophaena affinis* |  | SF |  | 1 |  | 1 | 2 |
| *Gyrophaena angustata* |  | SO |  | 3 |  |  | 3 |
| *Gyrophaena strictula* |  | SO |  | 53 |  |  | 53 |
| *Hadreule elongatula* |  | SO |  |  | 1 | 1 | 2 |
| *Hadrobregmus pertinax* |  | SO |  | 46 | 1 | 3 | 50 |
| *Hallomenus binotatus* |  | SO |  | 1 | 7 | 7 | 15 |
| *Haploglossa villosula* |  | SF |  | 1 | 1 |  | 2 |
| *Harminius undulatus* | 3RL | SO | NT | 8 | 8 | 10 | 26 |
| *Holobus flavicornis* |  | SF |  |  | 3 |  | 3 |
| *Hylastes brunneus* |  | SO |  |  | 2 | 3 | 5 |
| *Hylastes cunicularius* |  | SO |  | 385 | 430 | 345 | 1160 |
| *Hylastes opacus* |  | SO |  | 1 |  |  | 1 |
| *Hylecoetus dermestoides* |  | SO |  |  | 2 | 1 | 3 |
| *Hylobius abietis* |  | SO |  | 57 | 12 | 10 | 79 |
| *Hylobius piceus* |  | SO |  | 1 | 1 |  | 2 |
| *Hylobius pinastri* |  | SO |  | 1 |  |  | 1 |
| *Hylurgops glabratus* | 6 | SO |  | 80 | 833 | 588 | 1501 |
| *Hylurgops palliatus* |  | SO |  | 167 | 672 | 851 | 1690 |
| *Ips typographus* |  | SO |  | 84 | 82 | 225 | 391 |
| *Ischnoglossa elegantula* |  | SF |  | 44 | 215 | 164 | 423 |
| *Lacon conspersus* |  | SO | NT | 2 |  |  | 2 |
| *Lacon fasciatus* | 2RL | SO | NT | 32 |  |  | 32 |
| *Lasconotus jelskii* |  | SO | VU | 4 |  | 4 | 8 |
| *Latridius brevicollis* |  | SO |  |  | 1 | 2 | 3 |
| *Latridius consimilis* |  | SF |  | 5 | 1 | 3 | 9 |
| *Latridius hirtus* |  | SO |  |  | 1 | 2 | 3 |
| *Latridius nidicola* |  | SF |  |  | 1 | 1 | 2 |
| *Leptusa fumida* |  | SO |  | 8 | 13 | 10 | 31 |
| *Leptusa pulchella* |  | SO |  | 9 | 40 | 31 | 80 |
| *Liogluta microptera* |  | SF |  |  | 2 | 2 | 4 |
| *Liotrichus affinis* |  | SF |  | 1 | 2 | 9 | 12 |
| *Lordithon lunulatus* |  | SF |  | 8 | 3 | 6 | 17 |
| *Lordithon speciosus* |  | SO |  | 2 | 7 | 12 | 21 |
| *Lordithon thoracicus* |  | SF |  | 2 |  | 2 | 4 |
| *Lygistopterus sanguineus* |  | SO |  | 2 |  |  | 2 |
| *Magdalis frontalis* |  | SO |  |  | 1 |  | 1 |
| *Malthinus biguttatus* |  | SO |  |  | 1 |  | 1 |
| *Malthinus punctatus* |  | SO |  |  | 1 |  | 1 |
| *Malthodes brevicollis* |  | SO |  | 7 | 9 | 10 | 26 |
| *Malthodes fuscus* |  | SO |  | 23 | 22 | 16 | 61 |
| *Malthodes guttifer* |  | SO |  | 10 | 32 | 36 | 78 |
| *Malthodes marginatus* |  | SO |  | 1 | 9 | 3 | 13 |
| *Malthodes minimus* |  | SO |  | 1 |  |  | 1 |
| *Megasternum obscurum* |  | SF |  |  |  | 1 | 1 |
| *Megatoma undata* |  | SF |  | 2 |  |  | 2 |
| *Melanotus castanipes* |  | SO |  | 33 | 46 | 16 | 95 |
| *Microscydmus minimus* |  | SO |  | 1 |  |  | 1 |
| *Mniusa incrassata* |  | SF |  | 1 |  |  | 1 |
| *Molorchus minor* |  | SO |  | 4 | 1 | 3 | 8 |
| *Monochamus sutor* |  | SO |  | 79 | 1 |  | 80 |
| *Monochamus urussovi* |  | SO | EN |  |  | 2 | 2 |
| *Mycetochara obscura* |  | SO | NT | 1 |  |  | 1 |
| *Mycetophagus fulvicollis* |  | SO | NT |  |  | 2 | 2 |
| *Mycetoporus rufescens* |  | SF |  |  | 1 | 2 | 3 |
| *Nepachys cardiacae* |  | SO |  | 14 |  |  | 14 |
| *Nevraphes coronatus* |  | SF |  | 2 | 10 | 4 | 16 |
| *Nudobius lentus* |  | SO |  | 156 | 12 | 18 | 186 |
| *Olisthaerus megacephalus* |  | SO | NT |  | 1 | 2 | 3 |
| *Olisthaerus substriatus* | 10RL | SO | NT |  | 3 | 4 | 7 |
| *Omalium caesum* |  | SF |  |  | 1 | 1 | 2 |
| *Omalium rugatum* |  | SF |  | 3 | 11 | 5 | 19 |
| *Orchesia micans* |  | SO |  |  |  | 1 | 1 |
| *Orthocis alni* |  | SO |  | 2 | 2 |  | 4 |
| *Orthoperus atomus* |  | SF |  | 4 | 15 | 12 | 31 |
| *Orthoperus nigrescens* |  | SO |  |  | 3 |  | 3 |
| *Orthoperus punctatus* |  | SF |  |  |  | 2 | 2 |
| *Orthotomicus laricis* | 2 | SO |  | 2054 | 145 | 535 | 2734 |
| *Orthotomicus proximus* |  | SO |  | 18 |  | 5 | 23 |
| *Orthotomicus suturalis* | 7 | SO |  | 542 | 4 | 15 | 561 |
| *Ostoma ferruginea* |  | SO |  | 2 | 1 | 9 | 12 |
| *Othius lapidicola* |  | SF |  |  | 1 |  | 1 |
| *Othius myrmecophilus* |  | SF |  |  | 2 |  | 2 |
| *Oxymirus cursor* |  | SO |  | 3 |  | 3 | 6 |
| *Oxypoda alternans* |  | SF |  | 6 | 6 | 4 | 16 |
| *Oxypoda annularis* |  | SF |  |  |  | 1 | 1 |
| *Oxypoda hansseni* |  | SF |  |  | 3 | 5 | 8 |
| *Oxypoda skalitzkyi* |  | SF |  | 39 | 69 | 64 | 172 |
| *Oxypoda soror* |  | SF |  | 2 | 1 | 1 | 4 |
| *Pediacus fuscus* |  | SO |  | 6 |  | 1 | 7 |
| *Phloeonomus monilicornis* |  | SO |  | 5 | 10 | 10 | 25 |
| *Phloeonomus planus* |  | SO |  | 16 | 11 | 16 | 43 |
| *Phloeonomus punctipennis* |  | SO |  | 4 | 19 | 13 | 36 |
| *Phloeonomus pusillus* |  | SO |  | 12 | 28 | 24 | 64 |
| *Phloeonomus sjoebergi* |  | SO |  | 313 | 1162 | 1070 | 2545 |
| *Phloeopora concolor* |  | SO |  | 1 |  |  | 1 |
| *Phloeopora corticalis* |  | SO |  | 2 |  | 1 | 3 |
| *Phloeopora testacea* |  | SO |  |  | 1 | 1 | 2 |
| *Phloeotribus spinulosus* |  | SO |  |  | 4 | 1 | 5 |
| *Phosphuga atrata* |  | SF |  | 1 |  |  | 1 |
| *Phyllodrepa linearis* |  | SO |  | 10 | 24 | 54 | 88 |
| *Phyllodrepa melanocephala* |  | SO |  | 1 | 1 |  | 2 |
| *Pissodes gyllenhalii* |  | SO |  | 10 | 42 | 5 | 57 |
| *Pissodes harcyniae* |  | SO |  |  | 3 | 10 | 13 |
| *Pityogenes bidentatus* |  | SO |  | 28 |  | 2 | 30 |
| *Pityogenes chalcographus* | 1 | SO |  | 9948 | 353 | 856 | 11157 |
| *Pityogenes quadridens* |  | SO |  | 2 |  |  | 2 |
| *Pityophagus ferrugineus* |  | SO |  | 6 | 2 | 3 | 11 |
| *Pityophthorus lichtensteinii* |  | SO |  |  | 1 |  | 1 |
| *Placusa atrata* |  | SO |  | 1 | 2 | 7 | 10 |
| *Placusa cribrata* |  | SO |  |  |  | 1 | 1 |
| *Placusa depressa* |  | SO |  | 1 | 11 | 8 | 20 |
| *Placusa incompleta* |  | SO |  |  | 19 | 16 | 35 |
| *Placusa tachyporoides* |  | SO |  | 2 | 20 | 13 | 35 |
| *Platycerus caprea* |  | SO |  | 1 |  |  | 1 |
| *Platysoma angustatum* |  | SO |  | 11 |  |  | 11 |
| *Platysoma minus* | 4RL | SO | NT | 11 |  |  | 11 |
| *Plegaderus vulneratus* |  | SO |  | 33 | 19 | 37 | 89 |
| *Pogonocherus decoratus* |  | SO |  | 2 | 1 |  | 3 |
| *Pogonocherus fasciculatus* |  | SO |  | 5 |  | 1 | 6 |
| *Polygraphus poligraphus* |  | SO |  | 3 | 298 | 5 | 306 |
| *Polygraphus punctifrons* |  | SO |  | 38 | 778 | 252 | 1068 |
| *Polygraphus subopacus* |  | SO |  | 30 |  | 2 | 32 |
| *Proteinus brachypterus* |  | SF |  | 1 | 6 | 4 | 11 |
| *Pteryngium crenatum* |  | SO |  | 1 | 7 | 8 | 16 |
| *Pteryx splendens* |  | SO |  |  | 1 |  | 1 |
| *Pteryx suturalis* |  | SO |  | 4 | 9 | 8 | 21 |
| *Ptiliolum caledonicum* |  | SO |  |  | 3 | 10 | 13 |
| *Ptinella aptera* |  | SO |  | 1 | 1 | 1 | 3 |
| *Ptinella johnsoni* |  | SO |  | 1 | 3 | 1 | 5 |
| *Ptinella limbata* |  | SO |  | 1 | 2 | 7 | 10 |
| *Ptinella microscopica* |  | SO |  |  |  | 1 | 1 |
| *Ptinus subpilosus* |  | SO |  | 5 | 5 | 11 | 21 |
| *Ptinus villiger* |  | SF |  |  |  | 1 | 1 |
| *Pytho depressus* |  | SO |  | 19 | 1 | 8 | 28 |
| *Quedius brevis* |  | SF |  | 3 |  |  | 3 |
| *Quedius cruentus* |  | SF |  |  | 1 |  | 1 |
| *Quedius fuliginosus* |  | SF |  | 1 | 2 | 5 | 8 |
| *Quedius maurus* |  | SO |  |  | 3 |  | 3 |
| *Quedius mesomelinus* |  | SF |  |  | 2 | 1 | 3 |
| *Quedius plagiatus* |  | SO |  | 120 | 515 | 383 | 1018 |
| *Quedius tenellus* |  | SF |  | 2 | 63 | 12 | 77 |
| *Quedius xanthopus* |  | SF |  | 1 | 19 | 7 | 27 |
| *Rhagium inquisitor* |  | SO |  | 36 | 3 | 6 | 45 |
| *Rhizophagus dispar* |  | SF |  | 63 | 341 | 278 | 682 |
| *Rhizophagus ferrugineus* |  | SO |  | 3 | 41 | 34 | 78 |
| *Rhizophagus grandis* |  | SO |  | 1 |  |  | 1 |
| *Rhizophagus nitidulus* |  | SO |  | 2 | 34 | 16 | 52 |
| *Rhizophagus parvulus* |  | SO |  | 4 | 5 | 6 | 15 |
| *Rhyncolus ater* |  | SO |  | 28 | 178 | 154 | 360 |
| *Rhyncolus sculpturatus* |  | SO |  | 8 | 62 | 63 | 133 |
| *Salpingus ruficollis* |  | SO |  | 1 | 3 |  | 4 |
| *Scaphisoma agaricinum* | 8 | SF |  | 581 | 11 | 62 | 654 |
| *Scolytus ratzeburgi* |  | SO |  | 2 |  |  | 2 |
| *Selatosomus impressus* |  | SF |  | 6 | 2 |  | 8 |
| *Sepedophilus constans* |  | SF |  |  |  | 1 | 1 |
| *Sepedophilus immaculatus* |  | SF |  | 7 | 6 | 14 | 27 |
| *Sepedophilus littoreus* |  | SF |  | 117 | 316 | 284 | 717 |
| *Sepedophilus testaceus* |  | SF |  | 3 | 1 | 2 | 6 |
| *Silvanus bidentatus* |  | SO |  |  | 1 |  | 1 |
| *Soronia grisea* |  | SO |  | 2 |  |  | 2 |
| *Sphindus dubius* |  | SF |  | 6 | 1 |  | 7 |
| *Stenichnus bicolor* |  | SF |  | 17 | 44 | 51 | 112 |
| *Stenotrachelus aeneus* |  | SO |  | 4 |  |  | 4 |
| *Stephostethus rugicollis* |  | SF |  | 2 | 1 | 1 | 4 |
| *Tachinus subterraneus* |  | SF |  |  |  | 1 | 1 |
| *Tachyta nana* | 9RL | SO | NT | 3 |  |  | 3 |
| *Tetropium castaneum* |  | SO |  | 40 | 98 | 44 | 182 |
| *Tetropium fuscum* |  | SO |  | 8 | 41 | 11 | 60 |
| *Thanasimus femoralis* |  | SO |  | 1 | 1 | 10 | 12 |
| *Thanasimus formicarius* |  | SO |  | 14 | 3 | 5 | 22 |
| *Thyasophila wockii* |  | SO |  | 3 | 1 | 3 | 7 |
| *Trichophya pilicornis* |  | SF |  | 1 | 2 | 5 | 8 |
| *Triplax russica* |  | SO |  | 1 |  |  | 1 |
| *Trypodendron domesticum* |  | SO |  | 1 | 8 |  | 9 |
| *Trypodendron laeve* |  | SO |  | 9 | 3 | 10 | 22 |
| *Trypodendron lineatum* | 5 | SO |  | 81 | 1627 | 1457 | 3165 |
| *Trypodendron signatum* |  | SO |  | 1 | 3 |  | 4 |
| *Tyrus mucronatus* |  | SF |  | 15 |  |  | 15 |
| *Xantholinus tricolor* |  | SF |  | 1 | 1 | 3 | 5 |
| *Xylechinus pilosus* |  | SO |  | 1 | 2 | 10 | 13 |
| *Xylita laevigata* |  | SO |  | 38 | 2 |  | 40 |
| *Zilora ferruginea* | 6RL | SO | NT | 4 | 11 | 4 | 19 |
| *Zyras humeralis* |  | SF |  | 2 | 23 | 11 | 36 |
| Total abundance |  |  |  | 27457 | 24971 | 25758 | 78186 |
| Total species number |  |  |  | 279 | 266 | 263 | 372 |
| No of unique species (red-listed) |  |  |  | 57(5) | 25(2) | 26(5) |  |
|  |  |  |  |  |  |  |  |
